# Designing and Implementing an Ambulatory Oncology Nursing Peer Preceptorship Program: Using Grounded Theory Research to Guide Program Development

**DOI:** 10.1155/2012/451354

**Published:** 2012-05-09

**Authors:** Linda C. Watson, Shelley Raffin-Bouchal, Amy Melnick, Darlene Whyte

**Affiliations:** ^1^Department of Interdisciplinary Practice, Community Oncology, Alberta Health Services-Cancer Care, Calgary, AB, Canada T2S 3C3; ^2^Department of Graduate Studies, Faculty of Nursing, University of Calgary, Canada T2N 1N4; ^3^Nursing Education Department, Tom Baker Cancer Centre, Alberta Health Services-Cancer Care, Calgary, AB, Canada T2N 4N2

## Abstract

Having enough staff to provide high-quality care to cancer patients will become a growing issue across Canada over the next decades. Statistical predictions indicate that both the number of new diagnoses and the prevalence of cancer will increase dramatically in the next two decades. When combining these trends with the simultaneous trend toward health human resource shortage in Canada, the urgency of assuring we have adequate staff to deliver cancer care becomes clear. This research study focuses directly on oncology nurses. Guided by the grounded theory methodology, this research study aims to formulate a strategic, proactive peer preceptorship program through a four-phased research process. The goal of this research is to develop a program that will support experienced staff members to fully implement their role as a preceptor to new staff, to facilitate effective knowledge transfer between experienced staff to the new staff members, and to assure new staff members are carefully transitioned and integrated into the complex ambulatory cancer care workplaces. In this article, the data from the first phase of the research project will be explored specifically as it relates to establishing the foundation for the development of a provincial ambulatory oncology nursing peer preceptorship program.

## 1. Introduction

Cancer carries a sense of urgency in our modern world. In fact, it is a worldwide problem that is growing in unprecedented dimensions [1]. Globally, cancer is the world's most deadly disease [2]. In 2009, it was estimated that over 171,000 Canadian's faced a new diagnosis of cancer and a staggering 75,300 Canadians died of cancer [3]. The growing number of individuals diagnosed with cancer is primarily related to the increasing number of aging Canadians. Based on current trends, it is estimated that 40% of Canadian women and 45% of Canadian men will develop cancer during their lifetime. Further, it is estimated that 24% of women and 29% of men, or approximately 1 out of every 4 Canadians, will die from cancer [3]. Not only are cancer rates increasing, so are the number of Canadians living with cancer. According to statistics released in 2011 by the Canadian Cancer Society, there were over 749,000 cancer survivors in Canada at the beginning of 2007 [4]. This increase in prevalence is related to increasingly successful new treatments which are able to control progression. As a result, a continually escalating number of Canadians each year will be living with cancer as an ongoing health issue [5]. Given these realities, we will begin by addressing the impact this will have on the delivery of cancer care.

## 2. Current State of Knowledge

Just as cancer is a global crisis, so too is the shortage of health human resources. According to the World Health Organization (WHO), “The most critical issue facing the health care system is the shortage of people who make them work” [1]. Many significant reports in Canada have built momentum towards the prioritization of health human resources (HHRs) planning [6–8]. In 2005, a joint paper was published by the Canadian Nursing Association (CNA) and the Canadian Medical Association (CMA) titled *Towards a Pan-Canadian Planning Framework for Health Human Resources *[9]. Ten key principles and strategic directions for health human resource management were identified around three major themes: patient centered care, planning, and career life cycle. Specifically, the themes of planning and career life cycle identified strategic directions that revolved around improving the workplace environment and integrating preceptorship and mentorship across the career life cycle to support the continual professional development of staff.

Within the field of cancer care, HHR has also been identified as a priority. In 2007, the Canadian Partnership against Cancer (CPAC) was created as an arms length not-for-profit corporation to manage the implementation of a national cancer control strategy developed by a coalition of stakeholders [10]. The vision of this national group was to create a comprehensive, coordinated, high-quality person-focused cancer system that responds to the full range of needs of all Canadians and their families through all stages of the cancer experience [11]. The Institute of Medicine has defined high-quality care as care that is safe, effective, person-centred, timely, efficient, and equitable [12]. Science and medicine may make it possible to cure and, or treat cancer, but only people make cancer care a reality [13]. The issue of ensuring that there are enough well-educated healthcare professionals available to work within cancer care is fundamentally linked to providing high-quality cancer care now and in the future.

It is well understood that the oncology nurse's specialized knowledge is vital to effective care delivery across the trajectory of each individual's cancer experience [14]. As in other areas, nurses in cancer care act as a significant surveillance mechanism, identifying impending health crises, and intervening to limit the severity of the event [15]. As the number of cancer patients requiring care is expected to grow, and as the majority of cancer care is now delivered within the ambulatory setting [16], ensuring that the oncology nurses who work within the ambulatory setting are effectively supported to play their vital role in caring for and improving outcomes for these patients and their families is essential. As well, it is imperative that adequate staff are recruited and efforts are undertaken to retain staff within the ambulatory oncology setting [14]. Therefore, it has been concluded that the area of HHR planning in oncology nursing is a priority area of investment [10].

Within our provincial cancer care organization, the intersection of cancer trends and health human resource shortages moved from a theoretical level to the practice level after the development of a Provincial Nursing Council (PNC) in 2008. The PNC was created to allow oncology nurses from various roles and levels of the provincial cancer care agency to gather to discuss and provide leadership on issues that affect oncology nurses and their workplaces, and to guide the transformation and evolution of the cancer care system within our province. At the first PNC workshop, three key provincial areas of need were identified which included (1) a need to create a strategic direction for Oncology Nursing, (2) focusing purposeful attention on keeping and attracting the right people to oncology nursing, (3) and building a healthy work environment where oncology nurses are engaged in a process of continual professional growth and development. It was decided by the group that committing to and creating a provincial mentorship program was the most effective approach to address these issues.

The first step towards creating a provincial mentorship project was to explore the theoretical concepts of mentorship. Mentorship and preceptorship are key approaches that have been identified in numerous documents as holding strategic importance in ensuring adequate health human resources exists across the system [9–11, 17–26]. Although precepting and mentoring are often used interchangeably in the literature [27–29], there is some differentiation in the use of the terms in different geographical locations such as the UK and North America [29–31]. Both explain ways of using role modeling to support the professional growth of nurses, and to promote the overall quality of the practice environments, but are also unique in the focus and functions they serve [21]. Within this article, the term preceptorship is being defined as the means of transitioning new nurses into the workplace [32]. A preceptor is assigned to facilitate the new staff member's learning goals for a predetermined time, which is often short term. The focus of the relationship is the development of the new staff's clinical knowledge and skills relevant to the new work environment they are entering. Preceptorship usually occurs during regular working hours and can evolve into a mentorship relationship if both parties choose. Within the article, mentorship is being defined as a relationship that focuses on supporting continual professional development of existing staff members rather than on the transfer of skills [32]. A mentor is chosen or selected by the mentee. The relationship can span an extended period of time, sometimes years, and the focus of the relationship is to support professional growth and development. Although mentorship is also a nurturing, role modeling relationship, it is usually done away from the clinical setting [33].

**Figure 1:**
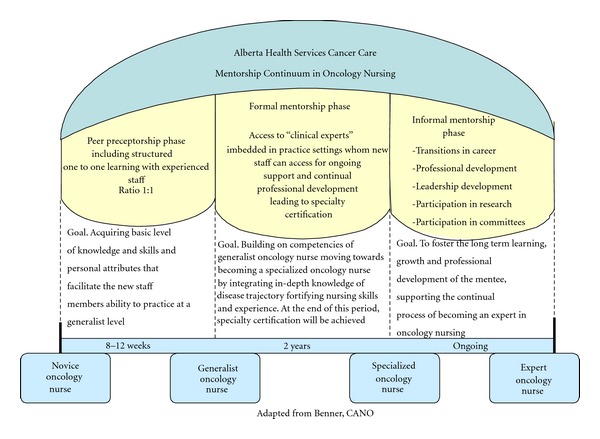
Conceptual model of mentorship as overarching principle.

Patricia Benner's “novice to expert” theory [34] and the Canadian Association of Nurses in Oncology (CANO) conceptual framework [35] were especially impactful to the group's thinking around this topic. Our group began to conceptualize mentorship as an umbrella concept that holds a trajectory of professional development over a series of distinct phases spanning the progression from novice to expert (see Figure 1). Further, the team conceptualized that within each distinct phase of professional development, forward progress needs to be supported by a specific type of constructive, nurturing relationship, with the peer preceptorship relationship between a new staff member and an experienced oncology nurse being the starting point on the continuum. As demonstrated within our conceptual framework, once the initial competency of the new staff member is achieved, the continual professional development needs to be supported by a mentoring relationship, more specifically, initially a formal mentorship relationship, in which the action of the relationship is focused on assuring the new staff member achieves oncology specialization. After specialization is achieved, the purpose of the mentoring relationship shifts to a more informal relationship that is driven by context or professional development. This framework was endorsed by PNC, and the initial area of peer preceptorship was approved as being the essential starting point for designing a change initiative, although the group indicated that the whole framework was needed. The overarching goals of continuum were established as (1) to assist the new staff member to move along the continuum from novice oncology nurse to expert oncology nurse, and (2) to offer professional development, support continued learning, personal growth, and leadership skills in existing staff.

It is important to note that the team selected the phrase “peer preceptorship” to differentiate the work of experienced staff with new staff, from the work of experienced staff with student nurses. Although there are many similarities between the work that preceptors do in both roles, the fact that work place competence drives the peer preceptor relationship is an essential core concept to this trajectory approach to mentorship. Historically in nursing, peer preceptorship has been taken for granted. Many programs exist to support staff nurses to precept students, and it is standard within our provincial nursing union that staff nurses receive additional pay for precepting students [36], but there is no such additional pay for precepting new staff to the workplace. In many workplaces, it is the same staff nurses who have received the preceptorship training to work with students who then are used to precept new staff, because they have the developed skills. However, within the ambulatory oncology nursing setting, very few student nurses do practicums due to the advanced skills and competencies necessary in this setting. Due to this limited exposure to student precepting the ambulatory oncology nurses are lacking formalized education to effectively transition new staff into the workplace. Although it is standard practice to “buddy” a new staff member with an experienced nurse until they achieve an adequate level of competency, this “buddy” relationship has received minimal formal support. An assumption has been made that if an experienced oncology nurse is good with his/her patients, he/she will be good at precepting new staff [37]. Currently within our provincial cancer care agency, no standardized program exists for supporting the preceptor in actualizing and being effective in the role of preceptor. This has resulted in differences in support and approaches to preceptorship across the province.

Staff, educators, and managers within oncology nursing settings across the province have indicated both an interest and a pressing need for additional support around the development and standardization of preceptor skills in existing staff and improvement in how new nursing staff are transitioned into and supported in cancer care workplaces across the province. As a response to this gap, this research study was designed to contribute to the development of a provincial approach to both preparing preceptors and supporting the preceptorship phase of transitioning new staff into their ambulatory oncology nursing roles. In terms of the conceptual framework, two areas of professional development are being addressed in this initiative (1) transitioning the novice nurse into their new role as an oncology nurse, and (2) informal mentorship for the experienced staff member to build their skills and competencies for being a preceptor. Ethical approval for this study was obtained from the local Conjoint Health Research Ethics Board.

## 3. Method

This study has been informed by grounded theory (GT) methodology as described by Glaser [38]. GT is defined as “the discovery of theory from data systematically obtained from research” (page 2). The goal of GT is to “generate a theory that accounts for a pattern of behavior which is relevant and problematic for those involved” [39] (page 93). This kind of qualitative inquiry facilitates the discovery of rich descriptions from which patterns of behaviors and descriptions of unique experiences can be gathered from those experienced with the phenomenon under discussion [40]. GT research differs from verification studies in that GT aims to discover theory through emergence instead of through hypothesis testing or verification. Verification studies are linear in design moving from research question, to sample selection, data collection, to analysis. GT research carries out these stages simultaneously [39]. Consequently, in the interview process, the questions are constantly evolving.

The distinguishing characteristics of GT are (1) the researchers attempt to uncover the basic social processes in the study setting instead of simply describing the behavior under study, (2) constant comparative analysis of each piece of data is underway throughout the data collection stage in order to identify emerging conceptual properties, and the relationships among the categories and their properties, (3) continual refinement of generalizations and a constant awareness of changes in the data collected as the study progresses and how that correlates to theoretical constructs that emerge, and (4) a theory results that is grounded in the data at hand [41].

**Figure 2:**
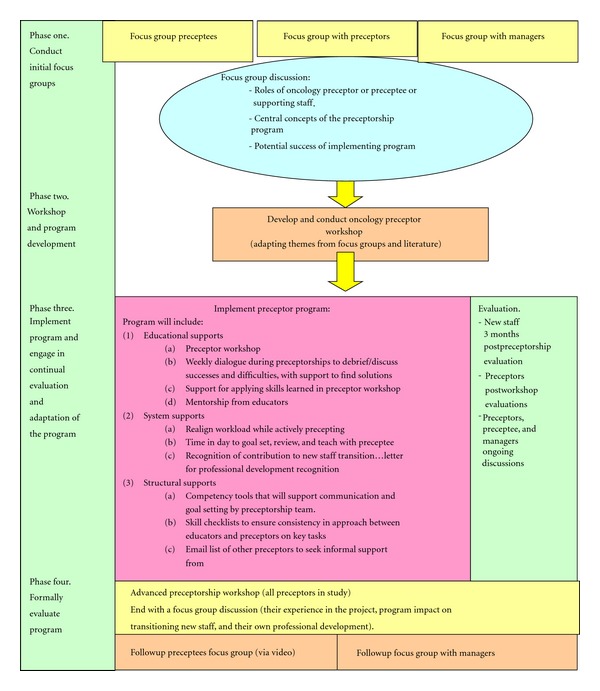
Schematic of ambulatory oncology peer preceptorship project.

The aim of GT is to understand how study participants see the world and how they define their problems as well as what would be helpful to resolve them. This method was selected to study preceptorship as it could assist the research team to uncover the processes involved in supporting existing staff and transitioning new staff across key groups. In grounded theory, a broad research question is the starting point, and through a process of discovery through individual and group dialogue, the researchers become sensitive to the questions that needed to be asked. Three broad research questions for this study were selected including: “What is the current process of nursing peer preceptorship in ambulatory oncology settings?,” “What, if any, components of the process need to be modified, added, or eliminated to facilitate the effectiveness of transitioning new staff into their oncology nursing role?,” and “What impact has the preceptorship program had on the preceptorship period?” This project has been designed in a phased approach (see Figure 2 for a schematic of the phased study design). At this point, only phase one is completed. In the first phase, we focused on the first two research questions in order to establish a broad understanding of preceptorship as it currently exists and to ascertain what innovations could improve this process. In phase two, this foundational knowledge will be synthesized with best evidence from the literature to develop the educational supports and peer preceptorship program that will then be implemented (phase three) and evaluated (phase four). The data from the initial focus groups (phase one) will be explored in this article.

**Figure 3:**
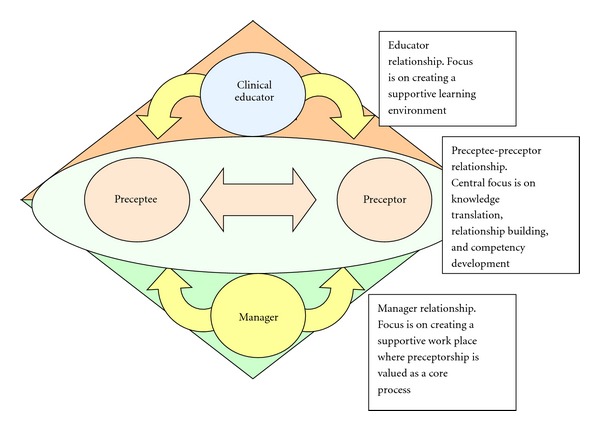
Schematic of relationships.

Preceptorship is a complex phenomenon which is essentially a relational process. There are four important relationships at work in preceptorship: the learner (preceptee), the hands on teacher (peer preceptor), the instructor (clinical educator), and the manager (supporter). These relationships vary in intensity and visibility during the preceptorship phase, but preceptorship cannot be enacted without the interplay of these interrelated roles (see Figure 3 for schematic). In order for the researchers to understand more fully the process of peer preceptorship, the perspective of all participants involved in the preceptorship relationship needed to be ascertained. The best approach to collect this data was to conduct in-person interviews or focus groups, however, due to the large geographical area of our provincial cancer agency, and in an effort to be cost effective, focus groups were conducted in a large urban center with rural members joining via video conferencing.

A purposeful approach to sampling was used [42]. Participants for the focus group discussions were invited by the research team on the basis of their ability to speak to the topic and contribute to a rich discussion. The research team contacted new staff members, past preceptors, and managers to discuss the project. All clinical educators from the sites were on the research team so therefore no specific focus group was held for educators. Separate focus groups were set up for each of the three groups. Informed consent was secured from participants prior to the focus group discussions. After introductions and ground rules were established, the following open-ended question initiated the focus group discussion, “What is your experience with peer preceptorship in your institution?” Other questions included “What key factors do you think would improve the peer preceptorship experience?,” “What specific skills do you think are essential for a positive experience within the peer preceptorship phase?,” “What kind of additional supports would be helpful in further developing these skills?,” “Can you share an example of specific challenges or frustrations that have played out for you in a past peer preceptorship experience?,” and “If you could give advice to the team starting up a new peer preceptorship program, what specifically would you recommend?” The focus groups were audio recorded and transcribed verbatim. Each focus group had between 6–10 participants. A facilitator (one of the investigators) guided the focus groups, and an observer (the primary investigator) recorded field notes based on the observations of individual responses and group dynamics. All field notes were part of the data and analyzed with the focus group transcriptions. Observations will continue with the second phase of the study.

## 4. Data Analysis

Glaser [38] contends that in GT all data is data. As the data was collected from these focus groups, the process of analysis began. The synchronicity of the process of data collection and analysis is one of the hallmarks of grounded theory with new pieces of data adding depth to the inquiry [43, 44]. By using a constant comparative approach in the data gathering and coding stages, the researchers actively build towards a theory that is grounded in the data. According to Glaser [43], GT is “the generation of emergent conceptualizations into integrated patterns, which are denoted by categories and their properties” (page 1). In this process of conceptualization, each data element is compared to every other data element as data are acquired. Data analysis began with the first focus group and continued throughout the first phase of the research.

The initial steps taken to accomplish this were for two members of the research team to individually and collectively code each transcript in sequential order at multiple levels of abstraction. We began with *in vivo* (open) codes line by line of participant's words and phases, to identify possible beginning coding categories (substantive coding) moving from raw verbatim data to abstract ideas and concepts until we were able to begin theorizing. Prior to each transcript being coded, both team members read the transcript in its entirety, and memos were created to clarify concepts and hypothesize connections between ideas, in keeping with grounded theory traditions. These memos also allowed us to identify and hold our preconceptions “in abeyance” in order to let the data speak for itself. The initial process of coding resulted in 174 codes for transcript number one, 175 codes for transcript number two, and 97 codes from transcript number three, for a total of 446 codes.

 These resulting codes were then broken down into data chunks (incidents) that were given labels known as categories or concepts. During open coding, the researchers broke the data down into incidents that we compared with one another for similarities and differences while asking the neutral question “what category or property does this incident indicate?” [39] (page 39). Incidents continued to be compared with other incidents until no other new incidents were found.

**Figure 4:**
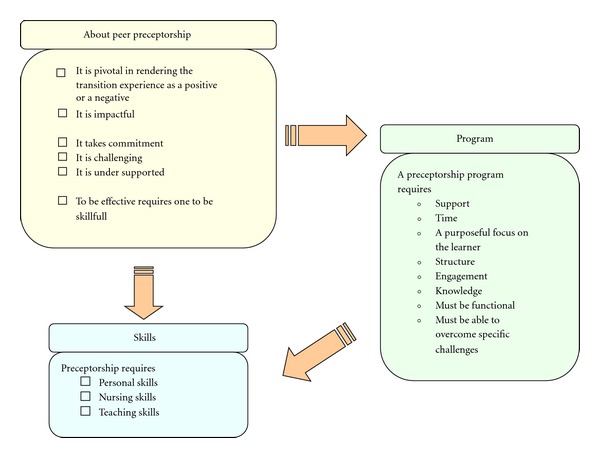
Three categories that emerged from the data.

Once all three transcripts were independently coded and compared in terms of each transcript, the next step was to collapse similar codes and categories across all three transcripts. This is known as selective coding. These categories are tied together through relational statements known as hypotheses. During the first phase of the research, three categories emerged: “About Peer Preceptorship,” “Skills Required,” and “Peer Preceptorship Program Considerations” (see Figure 4). As this is only the first phase of the study, and the purpose of the data collected is to inform the development of the program, the beginning core category was* Uncovering the Foundation and Identifying Strategies for Strengthening Peer Preceptorship in Ambulatory Oncology*. The resulting basic social process will not emerge until the final phase of the research.

### 4.1. Addressing Rigour

An important aspect of doing qualitative research is conducting the study and analysis with rigour. Criteria for evaluating the quality of grounded theory are generally described as* fit, workability, relevance, and modifiability* of the data [38, 42]. *Fit* means the codes, categories, and themes all fit with the data and are not forced by the researcher. In this study, fit was ensured by having two researchers code and categorize data independently, having a separate member of the research team examining the codes for appropriateness and inclusion of data, and by maintaining a clear audit trail indicating how field notes and memos were used, how data were reduced, and how decision points were reached.

*Workability* means that the theory is able to explain the phenomenon and to predict and interpret action under certain conditions. In other words, to determine workability is to determine if the theory works. For this study, the next phase of the research project will be where workability will be tested. The themes that were generated in the analysis will guide the formation of a peer preceptor workshop. At this workshop, the participants will be asked to review the themes and relationships identified. The workshop participants will also be asked to reflect on any areas that they feel are relevant to their ability to fulfill the role of peer preceptor that has not been covered in the workshop (member checks) to determine if the themes that emerged from the focus group discussions were reflective of the issue of peer preceptorship in its entirety.

Similarly, *relevance *of the research requires that the study deal with real concerns of the participants. This will be confirmed by the participants and others experiencing the same process. Relevance also conveys a sense of transferability; a high-quality grounded theory should hold meaning and significance for others in similar situations. The peer preceptorship workshop will be used to examine relevance of initial themes and to expand the emerging theory. The theory will continue to evolve over the next phase of the research which will consist of the implementation of a comprehensive peer preceptorship program. The question that will guide the evolution of the program will be “is this program relevant to the workplace setting and the issue of peer preceptorship.” Final focus group discussions will be held in Stage 4 of this research project to determine relevance of the final product with the uses of the program including preceptees, preceptors, and managers. As the clinical educators are part of the research team, their contributions to establishing relevance will be captured through their ongoing memos. Finally, the researchers will present the findings to oncology nurses who have been preceptors and to nurses at conference meetings inviting feedback in discussion periods.

A quality theory must be flexible enough to adapt to changing environments, and as such must be *modifiable. *As new data arise, current categories must be modified to incorporate them. A modifiable theory reflects quality and attendance to the data collection/analysis process. Through the audit trail, another researcher should able to see the relevance of new data to existing data.

## 5. Results

### 5.1. About Peer Preceptorship

In the focus groups, we did not define peer preceptorship, but instead, sought participants understanding of what it meant for them. It became evident that across all transcripts there was a common understanding that peer preceptorship is a pivotal period in the transition of new staff into a new workplace. How the peer preceptorship relationship played out has a direct impact on how that experience is remembered by the new staff and directly affects their desire to stay or leave their new place of work. One participant recalled how her preceptors were centered on her learning.

*“It was a valuable learning experience for me because they helped me with a lot of great learning experiences. We were a small center and…I had two preceptors that helped me master new learning opportunities and I also knew I had somebody else that was kind of looking out for me to help me advance in nursing and in my skills and so I found that very valuable as well.” *

If the experience is positive, the new staff member is left feeling that they are in a safe learning environment that is responsive to and supportive of their learning needs, leaving the new staff member with a sense of hope that they will be able to practice and grow in their oncology nursing skills and that access to ongoing support will be available via their preceptor even when the preceptorship phase is completed. The preceptor sets the tone by providing a safe space to ask questions, and helps with integration into the team by introducing the new staff to the work place and colleagues. Participants used words such as patience, grace, and respect to describe how they made them feel safe and comfortable in their learning.

*“I think for me it was just super positive in that they had a lot of grace for making mistakes or asking silly questions and for me that set the total tone of the entire time I was working here, so it was kind of a total positive safe place to learn and that still continues on.”*

On the other hand, when the experience is remembered as negative, it directly impacts the new staff members feelings of commitment and safety in their workplace, and in some cases participants spoke of situations in the past where they had left positions directly because of a poor preceptorship experience.

*“I had a preceptor at the hospital and I couldn't get out of there fast enough, I hated it—and part of it was that her nursing process was just horrendous—I just I felt like I wanted to be critical but the whole time I couldn't … I'm supposed to be learning um but I just was so disillusioned by what was going on—it was culture of go to coffee come back check the computer to see if the doctor had made any orders and I did one day kind of lose it and said you know I want to look at the patient—if the patient's dead we know what we're going to do and if the patient's got questions well you know and I was just- and the patient will tell us if the doctor's been. And I guess I um—just ran from that place. And they wanted me to come and work on the unit and I just said I can't—.” *

Being a peer preceptor is an important role that impacts both the new staff and the preceptor individually. It facilitates a sense of satisfaction knowing that one has contributed to someone else learning and that you have helped their socialization to the team. Participants spoke of how it is gratifying to expose the learner to new opportunities within the ambulatory oncology setting.

*“I also find it very satisfying. It's just such a different area to be a preceptor in as opposed to being on the in-patient side. I just find it very satisfying that you are giving a new staff member an opportunity that they might not necessarily have had before. You're giving them this completely different take on nursing and they get to see the out-patient side that you don't necessarily get to see elsewhere, so I really enjoy that part of it.”*

Peer precepting was recalled as enjoyable, influential, and offering preceptors a sense of pride. They indicated that being a peer preceptor demanded a commitment to one's own continual growth. Each new preceptorship relationship requires the preceptor to be patient, and to recognize that peer preceptorship is a reciprocal learning experience which might mean they will not have all the answers. The peer preceptor must be open to learning with the new staff member as well as teaching them.

*“We can go a long time without a certain protocol popping up or a certain situation so that I find I need to learn as much as the person who has not been here for very long, and if a situation comes up that doesn't happen for a few years then I need to review it as well so we can learn together … get the book or get on the computer and go over it together. It is not just about them learning it for the first time, it is me relearning it, because I have not seen it for a while.”*

The participants recognized that preceptoring has some negative impacts too, such as it adds challenges and complexity to their workday. “*You're trying to give someone a really thorough orientation and you do not want it to be rushed and yet you've got your work that still needs to be done by the time you go home so that been a little bit of a challenge.” *It takes time to precept new staff, and often there is little or no modification of workload to reflect this added responsibility. The findings revealed the importance of acknowledging that the learning curve is very steep for staff nurses who are new to ambulatory oncology but even more so when the new staff member has no oncology experience or is a recent graduate. Areas of particular challenge included conflict management, adapting teaching approach to the learner, encouraging reflective practice and critical thinking, helping the new staff set goals for learning, and balancing the new staff member's wish for more time to learn with the institutional push for completion of preceptorship on schedule.

*“In my experience with some of the new staff and preceptors I think across the board something you consistently hear is I would have liked more training, or I would have liked more time, like everybody wants more time to learn stuff and get immersed in the environment, but the clear expectation part is huge in that we need you to be functioning by X.” *

Participants acknowledged that there is currently no professional development support around being a preceptor and that often it is a trial and error approach to learning the required skills. The lack of mentorship for the preceptors contributes to the challenge of the role.

### 5.2. Skills Required

It was widely recognized across all three focus groups that being a preceptor requires the development of specific skills. The more one actively engages in being a preceptor, the more one's skills grow. Specifically, there was recognition among the groups that there were three distinct areas of skill development required to effectively precept a new staff. These include personal, nursing, and teaching skills. Excellent oncology nursing skills and knowledge of institutional policies and procedures are fundamental to the experienced staff's ability to precept the new staff. Currently, this domain within preceptor skill development is well developed through ongoing staff education.

Participants spoke of recognizing that effectiveness is enhanced if the preceptor has a highly developed cadre of personal skills. These include skills around communication, managing relationships, and assuming an approachable stance. “*Right away, when I started feeling welcomed it created a positive effect on my ability to learn and to know that I had support, so I think just being open and welcoming is important.”* One participant shared how her preceptor's relational skills relayed trust in her as a new learner in her initial time spent with her.

*“My preceptor that I was paired with would introduce me to her patients that they had been working with already so right off the bat that kind of conveyed this support and trust in me as somebody that they were going to be working with and I felt because of that support my patients kind of instantly trusted me and I feel like that had a huge part to do with our patient's acceptance of my working there as well.”*

The participants spoke of how important it is to be able to create a work/learning space where questions are encouraged, goals are mutually set, guidance is offered to achieve those goals, constructive feedback is delivered in a respectful manner, and where the learner is empowered to grow in skill and confidence. The notion of an approachable stance speaks to the personal approach that conveys self-confidence, while still being open to learning from the new staff member. It also speaks to being patient, encouraging, welcoming, and nonjudgmental.

*“I think it is about being comfortable to have them ask questions … and creating an environment where they are comfortable to ask the question … it is not so much about actually wording the questions well … I think its about the back and forth talking. I think when I say being encouraged to ask questions, it is more about comfort and familiarity and not being judged about one's level of intelligence or anything like that…”*

Currently, these personal skills are not being actively nurtured within the preceptor beyond their day-to-day experiences in delivering cancer care or through their own personal development activities.

The third area of skill development is teaching skills. An example is understanding and applying adult principles of learning and knowing how to adapt teaching approaches, or understanding how to assist the learner to find their own answers instead of just giving the answers. The participants spoke of the importance of understanding how to gradually transition from being the teacher to being the resource, and how to engage in mutual learning to facilitate competence. Consensus from the participants was that currently we do not actively offer educational supports that are intended to support the staff nurses teaching abilities beyond the more familiar application of patient teaching. One manager shared her perspective.

*“I wonder about some sort of education for preceptors so they understand what the expectations are for them and maybe some approaches to help learning for the preceptee and how to deal with issues when they come up and where to go for that sort of thing.”*

### 5.3. Peer Preceptorship Program Considerations

A large part of the focus group dialogue centered on ideas for the development of a peer preceptorship program, as that is the core objective of this project. Six areas were identified as key issues that the program must address. These included support, time, focus on the learner, structure, engagement, and functional flexibility.

#### 5.3.1. Support

Participants asserted that preceptorship is a unit issue, not just an individual issue. Even if someone is not precepting, there needs to be a mutual understanding of the expectation that other staff members will assume some component of the increased workload. Precepting adds workload to the preceptor, so others will need to shoulder an increased work load, even if they are not actively precepting.

*“The peer preceptorship program has to be supported by all staff, because it affects everyone … even those who don't choose to be in it as a peer preceptor, others need to be supportive and aware they will be required to pick up more work so that the preceptor can focus on teaching the new staff.”*

The issue of workload also needs to be considered by the management team in that their involvement in active redistribution of the patient load will establish the collaborative tone of the unit. Another idea for support that the participants identified is a forum where preceptors can learn together over time. This could be through team debriefing or through advanced preceptor skill development workshops. Participants identified that mentorship for the preceptor was a current gap. They felt that ensuring the preceptor had access to a mentor in regards to their growth as a preceptor was a way to ensure continual development of their skill set.

*“When I had some people orientating, it is so helpful to have someone else around that has done a lot more preceptoring than I have. She was helpful to me because she helped me learn how to teach. Usually I don't have anyone like that whom I can go to like that, so it was very helpful to have her. It would be great to have someone like that to go to all the time for support and ideas on how to teach better.”*

The creation of a formal preceptor workshop to support the development of the various skills required for effective preceptorship was also identified as an important new support.

*“Maybe some kind of paid prep type would be helpful for preceptors I mean um—just to give them some basic suggestions or coaching in terms of what they need to know, what the goals are and just to get them to know how to be the best preceptor that they can possibly be.”*

#### 5.3.2. Time

The need to recognize that preceptorship takes time was a dominant discourse across all groups. Importantly, it needs to be recognized that preceptorship takes time on both sides of the relationship. Participants spoke on a consistent basis of time as a barrier to effective precepting and for learning without feeling pressure.

*“For me, I think it is really more time … I think we have lots of teaching skills around and lots of experience in the nurses who have been here a long time, but the real problem is time, enough time to get things across to the new person, and give them your thoughts on how things are best done or whatever…”*

The learner needs time to acquire new knowledge, time to reflect on new knowledge and how it fits into existing experiences and knowledge, time to develop competencies in new tasks and procedures, and time to learn a new system of care delivery. On the other hand, the preceptor needs time to get to know the learner and their background, time to explain and teach, time to debrief with learner and with the clinical educator, time to reflect on their own learning and development, and time to plan how to support the new staff member's competency development. One manager voiced the following.

*“If there is a commitment to this … then the preceptor should be given a certain amount of protected time … to prepare themselves, to be able to sit with the new employee and go over some of the learning objectives they had in mind, this is what were going to do, this is what I want you to watch me do and that would be very challenging in the current environment if they were continuing their clinical assignment as it were. So protected time would be a big thing … that would probably encourage staff to take on this role.”*

Currently, participants felt that the various dimensions of time utilization required in preceptorship are not acknowledged by the system. The fact that workload is not routinely adjusted when a preceptor is precepting is evidence of this.

#### 5.3.3. Focus on the Learner

The need for a purposeful focus on the learner was a strong message throughout the discussion. Learners wanted their past experiences and knowledge to be recognized, valued, and taken into account.

*“I came into this new experience with already a background in oncology … so for me I think maybe having that taken into consideration a little bit would have been good. There was a lot of it was information that supposedly had to be reviewed with me but a lot of information I already knew and so we spent a lot of time reviewing basic information that was very familiar to me. So maybe, in my case recognizing that I was coming with the background of oncology already, to somehow be able to take that into consideration when it came to the actual content that needed to be delivered.”*

Understanding how the new staff member learns was also recognized as important, as then the preceptor could tailor their approach to supporting competency development. This category also held the notion that the preceptor had to pay attention to the learner, not just deliver information. Participants also acknowledged that the preceptor needs to be available to the learner, which relates back to the ability to adopt an open stance.

#### 5.3.4. Structure

Participants shared many ideas around structural components of a peer preceptorship program. Participants felt that matching the preceptor with the learner based on learning style and experience would be helpful. “*I think that matching is very important and while you may have very good preceptors for one, you may not be very good preceptors for others.”* They also indicated that clear expectations and objectives for both the learner and the preceptor for the preceptorship period would be helpful including the notion of gradually shifting the new learner's role from observing to doing while inversely shifting the preceptor's role from doing to observing. The participants discussed formalizing the collaboration and roles of the clinical educators and managers as both roles are fundamental to supporting the process of peer preceptorship. As demonstrated in Figure 3, the clinical educators are responsible for creating a supportive learning environment, while the managers must establish a supportive work environment.

#### 5.3.5. Engagement

Participants indicated that the only way a preceptorship program will work is if staff members want to be preceptors, and if new staff members want to become oncology nurses.

*“First of all, we have to be sure the preceptee is interested in being an oncology nurse. I mean sometimes it's just a job that has good hours, and they don't really want to be here. So confirming that there is a sincere interest in oncology nursing is essential.”*

It was recognized that somehow the program needs to inspire staff to want to be involved in preceptorship.

*“Currently there is low motivation and engagement among staff so this is an issue … no matter how wonderful a program you design, if you can't get your key staff to choose to participate in it or take the role of preceptor seriously it will not work.”*

Some ideas to enhance staff engagement discussed were securing financial compensation similar to working with students, letters of recognition of the preceptors contribution, backfilling shifts so preceptors can attend professional development opportunities, and ensuring a manageable workload while precepting. Even the act of being identified as someone who would make a good preceptor facilitates engagement. It was also discussed that it is essential that new staff members know what kind of nursing care they are being expected to provide. A preceptorship program will affect the whole nursing team, not just those who volunteer to precept, so it is important that broad education around this new conceptualization of preceptorship as a pivotal process is undertaken to ensure engagement of the entire team.

#### 5.3.6. Functional Flexibility

The participants spoke of needing a peer preceptorship program that was realistic. Fiscal responsibility demands that a newly developed peer preceptorship program be functional and flexible. The program must contribute to the timely and effective transitioning of new staff members into their roles, while supporting a high degree of safety and job satisfaction among both the new and experienced staff members. The program must also alert the educational and management team of situations where new staff members are not progressing as anticipated, so that proactive interventions can be designed to mitigate stress to all involved. However, the program must be flexible enough to adapt to the constant changes in the workplace, and issues that cannot be controlled for such as sick calls, staff shortages, and personality conflicts.

## 6. Discussion

### 6.1. The Benefits of Preceptorship

**Figure 5:**
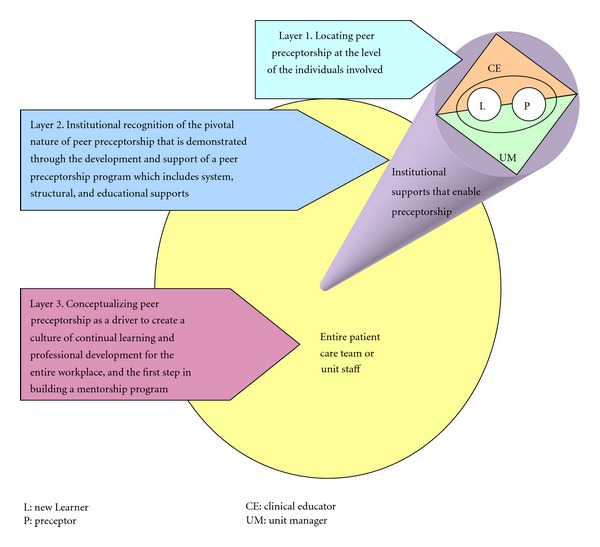
Conceptualizing the Relational layers of a Peer Preceptorship Program.

Findings suggest that the process of peer preceptorship involves layers of relational engagement (see Figure 5) beginning with the recognition that peer preceptorship is enacted at the level of the individuals involved in the process including managers, educators, preceptors, and the new staff members. The next relational layer is between the institution and those involved in peer preceptorship. There must be an institutional valuing of peer preceptorship as a skillful, pivotal process in continual professional development, retention, cost savings, job satisfaction, knowledge translation, and effective transitioning of new staff into the workplace. Institutional valuing must be demonstrated by the creation of supports that enable peer preceptorship and enhance the effectiveness and experience of being involved in peer preceptorship. The final relational layer of peer preceptorship is between the entire patient care team, or unit staff with those involved in peer preceptorship. The entire team must recognize that peer preceptorship is everyone's business even if one is not directly involved at the individual level. Peer preceptorship requires support from the entire workforce (see Figure 4 for a schematic representation). In the initial phase of this study, the process of understanding peer preceptorship as layered relational engagements has been conceptualized as *Uncovering the Foundation and Identifying Strategies for Strengthening Peer Preceptorship in Ambulatory Oncology*. The process was enacted in three overlapping ways: building an understanding of what preceptorship is *(about preceptorship), *the building of a skill set *(skills required),* and building the program that fits with the organization and all individuals engaging in the program (*program consideration*).

A plethora of literature supports the positive correlation between nurse preceptorship programs and nurse retention rates [21, 33, 45–47]. Preceptorship is also correlated with effective knowledge translation [17, 18, 21]. Further to this, evidence supports that increasing retention is associated with adequate and stable staffing, which directly affects patient safety issues such as medical errors, mortality, and average length of stay in an inpatient unit [48]. As well, increased retention decreases institutional costs by minimizing the need to hire and train new staff members [21, 46, 49, 50]. These positive indicators have spurred much interest in how the potential value of effective preceptorship can be integrated into healthcare organizations across the spectrum.

Within the research literature, a *recognized skill set* has been identified as being foundational in supporting the preceptor's ability to effectively guide the transitioning of new staff into their roles [51, 52]. This skill set includes enhanced communication skills (specifically around conflict management and how to give constructive feedback), adult principles of learning, how to set goals, how to manage conflict, motivation, managing diversity, socialization of new staff, and evaluation [17, 21, 33, 51]. It is important to note that the literature did not reveal research that explored the complexity of supporting ambulatory oncology nursing across a provincial organization.

Effective preceptorship can enhance the knowledge transfer from the experienced oncology nursing staff to the new staff member [53] to ensure safe, competent, and compassionate cancer care is delivered. The literature and the data from our study support the use of a preceptor workshop as effective to support the knowledge and skill development within the preceptor [17, 21, 23, 27, 54], but such support is only one aspect of a preceptorship program. Additionally, it has been well documented in the literature and has emerged from our data that numerous other supports are essential to facilitate successful peer preceptorships. These include

1. *system supports* such as managerial commitment to realign the workload/patient assignment given to a preceptor in order to free up some time and energy to actively focus on precepting the new staff member [23, 24, 55],
2. *educational supports* such as an ongoing dialogue with the educators around issues/successes encountered in the process of enacting the preceptor role [17, 23, 24, 33, 37, 54], and assuring the preceptors have access to a mentor to facilitate the continual development of their preceptor skill set [56]; building relationships has been highlighted as being of utmost importance as it is within a mutually supportive and respectful relationship that interactions that empower, inspire, guide, advise, and model clinical behaviors can be nurtured [57],
3. *structural supports* that facilitate continual progress toward successfully transitioning the new staff member into their specific role such as competency documents and check lists [18, 25, 33, 58].

Preceptorship is more than assigning a new staff member to “buddy” with an experienced nurse. The literature around this topic aligns well with the data that emerged from our focus group discussions. The researchers propose that approaching peer preceptorship as an integrated program that incorporates aspects noted within the literature and categories from our focus groups will maximize positive benefits to the work environment and ensure high-quality patient care outcomes.

## 7. Future Research

As stated earlier in this article, the three broad research questions for this study are: “What is the current process of nursing peer preceptorship in ambulatory oncology settings?,” “What, if any, components of the process need to be modified, added or eliminated to facilitate the effectiveness of transitioning new staff into their oncology nursing role?,” and “What impact has the preceptorship program had on the preceptorship period?” The data analysis reported here speaks to the first two research questions. The data supports a broad basic understanding of preceptorship as it currently exists within our provincial cancer care agency and clearly has identified what innovations could improve this process. This foundational knowledge will now be leveraged into the second phase of this study in which specific educational supports and preceptorship program will be developed. The data from the participants will be integrated with the best practice evidence available around how to enhance the process of peer preceptorship. Once the comprehensive program is designed, it will be implemented for 12 months and evaluated by those involved in the peer preceptorship program. The remaining stages of this research study will focus on exploring the third broad research question posed in this study, “What impact has the preceptorship program had on the preceptorship period?”.

## 8. Conclusion

Providing adequate care for the growing number of Canadians requiring Oncology care will become a mounting issue over the next decade. As both the incidence and prevalence of cancer increase, the cancer care delivery systems will be stretched to meet the growing needs. Due to these realities, we anticipate a growing requirement for more oncology nurses within ambulatory cancer programs. The anticipated national health human resource shortage will complicate our ability to staff our ambulatory cancer care facilities adequately. The resulting reality is that our system needs to retain as many nurses as possible, recruit adequate numbers of new staff from a shrinking pool of nurses, and effectively and efficiently transition new staff members into a growingly complex ambulatory cancer work place. This research study was driven by our provincial cancer care agencies awareness of these trends and by our desire to be proactive and responsive to current and future staffing pressures. According to grounded theory methodology, theorizing is ongoing and open to continual revision. In this article, the findings of the first phase of our grounded theory study were explored. The first phase was an essential first step in the process, as it allowed the research team to understand what was currently occurring in the peer preceptorship phase and identify what our staff members believe would improve the process. The research is moving into second phase of the study, where the development of the details of the program will be grounded in our staff's experiences, ideas, and requests. This phased approach will allow the staff members involved in preceptorship to inform ongoing program development. The best available evidence will be used to build upon and strengthen areas identified by our staff and to fully develop a provincial peer preceptorship program that will help our provincial cancer care agency to be prepared to care for cancer patients across our province now and in the future.

In utilizing these findings to guide practice change, the major contribution of this research project will be established. All system redesign and program development will be grounded in the ongoing experience and ideas for improvement of our staff. Exploring and improving how the changes and new supports impact peer preceptorship will improve our understanding of how a systematic program can contribute to the effective transition of new staff into this workplace. It will also allow the research team to maximize the supports offered to experienced oncology nurses who are so essential to the process of transitioning new staff into this highly complex and rapidly evolving workplace, while still being responsible to the actual cost effectiveness and functionality of the program. Linking this research to practice change and then evaluating the effectiveness of the change will allow for the generation of a theory that will account for the patterns of peer preceptorship which is relevant and meaningful for all those involved.
